# Estimating Time and Size of Bioterror Attack

**DOI:** 10.3201/eid1007.030632

**Published:** 2004-07

**Authors:** Johan Walden, Edward H. Kaplan

**Affiliations:** * Yale School of Management, New Haven, Connecticut, USA;; † Yale School of Medicine, New Haven, Connecticut, USA

**Keywords:** Bioterrorism, Bayesian Analysis, Bayesian Forecast, surveillance, anthrax, perspective

## Abstract

In the event of a bioterror attack, rapidly estimating the size and time of attack enables short-run forecasts of the number of persons who will be symptomatic and require medical care. We present a Bayesian approach to this problem for use in real time and illustrate it with data from a simulated anthrax attack. The method is simple enough to be implemented in a spreadsheet.

## Abstract 2

Time and size of possible bioterror event estimated in real time.

In the event of a bioterror attack, once the biologic agent has been determined, rapidly estimating the size and time of attack enables a forecast of the number of persons who will be symptomatic and will require medical attention over the days (and perhaps weeks) after the attack. Such a forecast could play a key role in determining the response effort required, e.g., surge capacity planning at hospitals, distributing vaccines or antimicrobial agents to the population, as appropriate (1,2). We refer to early knowledge of the size and time of an attack as situational awareness.

We present a Bayesian approach to the real-time estimation of the size and time of a bioterror attack, from case report data, that is simple enough to implement in a spreadsheet. The model assumes a single-source outbreak caused by a bioterror attack at a particular point in time. Although the model assumes that the infectious agent is not contagious, the analysis still holds for contagious agents until secondary infections have progressed to symptomatic cases. Thus, our model should prove valuable within the first incubation period after an attack has been detected for a contagious agent and for longer time periods in the event of a noncontagious agent. However, in the event of multiple attacks at different points in time or an attack with a rapidly progressing contagious agent, the problem becomes more difficult and similar to the use of back-calculation to recover the incidence of infection over time from symptomatic case reporting (3).

The key assumptions in our analysis are that the biologic agent used has been identified and that the probability distribution of the incubation time from infection through symptoms is known. The incubation time distribution for anthrax has been estimated by Brookmeyer and colleagues on the basis of the Swerdlovsk outbreak (4); data describing the incubation distribution for smallpox are summarized by Fenner et al. (5). Although smallpox is a contagious infection, historically the incubation time from infection through onset of symptoms is 7–17 days (5), a fact that renders our model applicable to smallpox for roughly 2 weeks after an outbreak or 1 week after the first observed cases (which is the shortest time until one would expect to see cases resulting from second-generation infections).

Likely ranges for the incubation times of other plausible bioterror agents are available at the Centers for Disease Control and Prevention’s bioterror Web site (6), in addition to sources in the literature. If the only information available regarding the incubation time for some agent is a likely range, then one approach to creating a distribution for use with our model is to assume that the range corresponds to a probability coverage interval from a plausible incubation time distribution (such as the lognormal) and match the parameters of the distribution accordingly.

We assume that the attack is detected through the appearance of infected persons with symptoms, and that as cases are identified, patient interview yields the approximate time at which symptoms appeared, a process which avoids the need for explicit estimates of reporting delay. Corrected as such, case reports provide two types of information. The number of cases observed provides a lower bound on the size of the attack. The specific timing of case reports also conveys information that can be better understood when filtered through the agent-specific incubation time distribution.

The mathematical details of our approach are described in the Appendix. We define the time origin as the instant when the first case (and hence the attack) is detected (though the time origin can be reassigned if case investigation indicates that a subsequently reported case had earlier symptoms). At the moment the attack is detected, consistent with Bayesian principles (7), we presume a prior probability distribution (henceforth, prior) governing the size of the attack. For any given attack size *n*, the time since the attack can be equated to the minimum of *n* independent incubation times (since the attack is detected by the first symptomatic case). As additional cases accumulate over time, the likelihood of observing cases at specific times is computed with standard methods. Given the data observed, application of Bayes rule enables estimation of the posterior distribution of both the size and time of attack, from which summary statistics such as the mean, standard deviation, and probability intervals of the attack size and time are easily estimated. Short-run forecasts of future cases are also easily achieved within this framework. We have developed an Excel spreadsheet (Microsoft Corp., Redmond, WA) for implementing this procedure.

As an example, we simulated an anthrax attack that infects 100 persons using the incubation time distribution for anthrax estimated from the Swerdlovsk outbreak (4). We assumed a broad prior that assigns equal likelihoods to attacks of different orders of magnitude from 1 to 10,000 (Appendix). Thus, attacks infecting 1–10, 11–100, 101–1,000, and 1,001–10,000 persons each have the same 25% probability of occurrence. With this prior, absent any data other than the first case observed at time 0, the estimated mean attack size is approximately 1,090.

Absent intervention, the 100 victims in this simulated attack would appear as case-patients in accord with Figure 1 (open dots). Using the methods shown in the Appendix, we report estimates of the attack size and the time of the attack based on the cumulative number of cases observed at the end of day 5 of the outbreak (where the time origin corresponds to the occurrence of the first observed case) (Table). At the end of day 1, the estimated mean attack size equals 850 (with a 95% probability interval ranging from 60 to 3,300). However, estimates approach the true value of 100 over time. Similarly, the estimates for the time of the attack improve from 1.1 days before the first case (estimated after day 1 of the outbreak) to 1.8 days before the first case is observed; the true time of attack is 1.8 days before the first case observed in the simulated outbreak (Figure 1).

**Figure 1:**
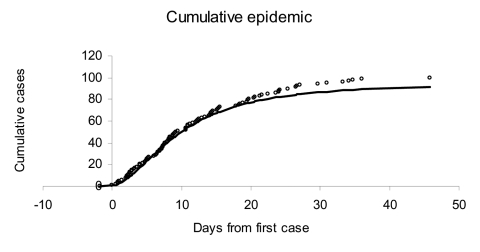
Simulated actual (open dots) and forecasted (solid curve) cumulative cases in an anthrax bioterror attack that infects 100 persons 1.8 days before the first symptomatic case is observed. The cases were simulated from a lognormal distribution with median 11 days and dispersion 2.04 days, which corresponds to the incubation time estimated for anthrax based on the Swerdlovsk outbreak (3).

**Table:** Real-time estimates of size and time of attack, given the total cases observed in the simulated outbreak^a^

Days past case no. 1	Total cases	Estimated attack size	Estimated day of attack (before case no. 1)
1	5	850	1.1
2	7	120	1.9
3	15	160	1.4
4	18	100	1.8
5	23	90	1.8

^a^See Appendix for details.

Figures 2 and 3 illustrate the posterior distributions of the initial attack size and time of attack at the end of 5 days (when a total of 23 cases have appeared). For example, Figure 2 suggests that while the expected attack size, given the data, equals 90, initial attacks as small as 50 or as large as 150 are also plausible. Similarly, the time of the attack could have been as recent as half a day before the first case was observed, or as early as 3.5 days before the first case appeared.

**Figure 2:**
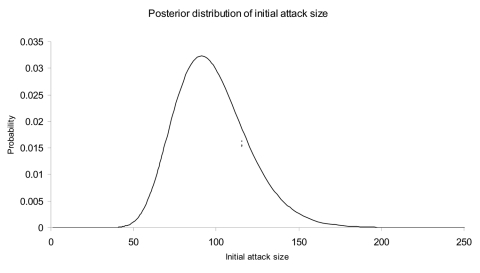
Posterior probability density of the attack size based on the data in Figure 1 observed through the end of day 5 after the first case appeared.

**Figure 3:**
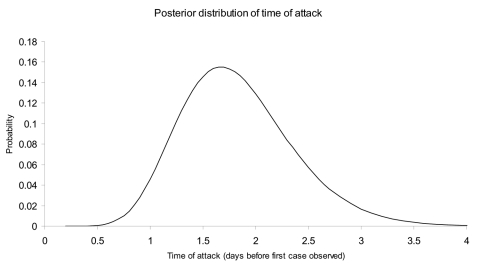
Posterior probability density of the time of attack based on the data in Figure 1 observed through the end of day 5 after the first case appeared.

Given estimates of the initial size and time of attack, one can forecast the occurrence of future cases over time, as shown in Figure 1 (solid curve), where the forecast is made on the basis of cases observed through the end of day 5 after the first case was observed. Such a short-range forecast could be helpful in determining the resources required to treat those infected in the attack, although once a widespread response to the attack is mounted (e.g., distribution of antimicrobial agents, in the case of anthrax), the forecasts lose their validity (8).

The key assumptions in our model are that the probability distribution of the incubation time from infection through development of symptoms is known and that attack victims can report the times of symptom onset (so we have not explicitly accounted for reporting delay). In an actual bioterror attack, determining the incubation time distribution itself might be necessary. For example, as shown recently by Brookmeyer et al. (9), the incubation time for anthrax is dose-dependent. Thus, exposure to anthrax powders with much greater spore concentrations than evident either in Swerdlovsk or the U.S. postal attacks could lead to shorter incubation distributions. While we are investigating statistical methods for this more general problem (J.T. wu, unpub. data), having a relatively simple tool is still helpful when the probability distribution of incubation times is presumed known and a single, point-source outbreak is suspected. Our model might also prove helpful for education and training exercises, in addition to use during an actual bioterror attack.

## Appendix

We seek to estimate the initial attack size and the time of the attack from observed cases of infection in real time. The case report data are the (reporting-delay corrected) times at which cases have been reported. We intend this model to be applied once an attack has been discovered and assume that the agent is noncontagious (or in the case of a contagious agent, that no secondary transmission has occurred) and that any interventions mounted (such as vaccination or the administration of antimicrobial agents) have not yet had any effect on the early case reporting data. We define *t_j_* to be the time at which the *j*th case is observed and define the origin as the time at which the first case is observed (so *t*_1_ = 0). The unknown time from the attack until the first case is observed is denoted by *A* > 0 (and thus the actual date of the attack is equal to –*A)*, while the unknown number of persons infected in the attack is denoted by *N* >1.

We treat *A* and *N* as random variables and assume that the attack is detected through the reporting of the first case at time *t*_1_ = 0. At the time the attack is detected, we quantify our beliefs regarding the size of the attack by the prior probability distribution _
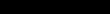
_. Let *X* denote the symptom-free incubation time for the attack agent, with probability density *f*(x) and survivor function _
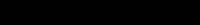
_ If *n* persons were actually infected in the attack, then the time from the attack until the first case is observed would equal the minimum of *n* independent incubation times_
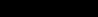
_ thus

_
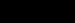
_
[1]

Consequently, the probability that *a* units of time would pass before the attack would be detected by the first case equals _
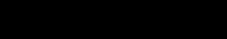
_from which the conditional probability density function of *A* given an attack of size *n* follows as

_
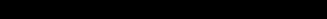
_
[2]

Equation 2 implies that the joint prior distribution for the size and time of attack when the first case is observed is equal to

_
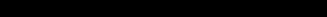
_
[3]

Now, suppose that by time *τ* an additional *k* – 1 cases have been observed at times _
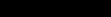
_ Conditional upon an attack of size *n* having occurred at time –*a*, the joint probability density of the data observed (that is, the likelihood function) is given by

_
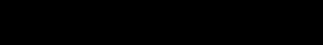
_
[4]

where **t** = *t*_2_, *t*_3_, *t*_4_,…, *t_k_*. Equation no. 4 is simply the conditional joint density of the first *k* – 1 order statistics observed from a sample of size *n* – 1, given that *a* time units had passed from the attack until the first case was observed at time 0, adjusted for the fact that the period of observation extends to time *τ* (10). Unconditioning the likelihood in equation no. 4 by the prior in equation no. 3 yields the joint density

_
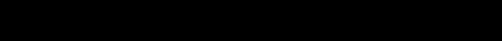
_
[5]

and application of Bayes rule yields the joint posterior distribution of the size and time of attack as

_
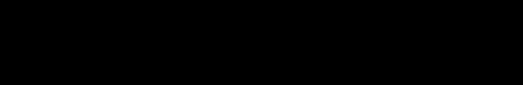
_ [6]

The posterior distributions of *A* and *N* are then easily obtained from equation no. 6 by summing (over *n*) or integrating (over *a*).

To obtain a short-run forecast of future cases, note that conditional upon an attack of size *n* that occurred *a* time units before detection, the expected number of cases that will occur by some future time τ* equals _
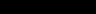
_. Unconditioning over equation no. 6 yields a simple short-run forecast of the number of future cases expected given all of the data observed to date. An even simpler approximation is obtained by substituting the posterior expected values of *N* and *A* in the expression above for the expected number of future cases; we used this approach in producing the forecast shown in Figure 1.

In our examples, we assume that, a priori, the logarithm of the attack size *N* is uniformly distributed between 0 and the logarithm of 10,000, and we approximate this distribution in a spreadsheet with 500 mass points equally spaced on the natural logarithmic scale. This procedure assigns equal probabilities to four different orders of magnitude, that is, attacks that infect 1–10, 11–100, 101–1,000, or 1,001–10,000 persons are each assigned the same 25% probability of attack. The expected prior attack size associated with this distribution approximately equals 9,999/ln(10,000) = 1,090.

Our examples also assume that the incubation time from infection through onset of symptoms is distributed in accord with a lognormal distribution with a median of 11 days and a dispersion of 2.04 days. This is the distribution fit to the data from the anthrax outbreak in Swerdlovsk (11). For numerical computations, this distribution is also approximated discretely within the spreadsheet.

**Figure A1:**
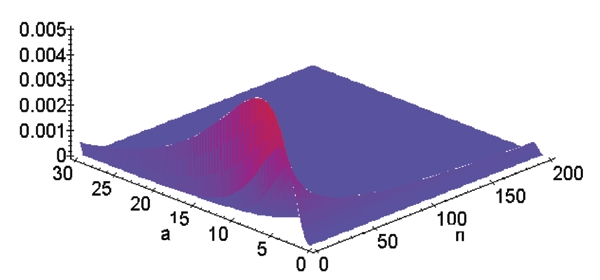
The joint prior distribution of the attack size and time of attack, based on the assumption that the logarithm of the attack size is uniformly distributed from 1 to 10,000.

**Figure A2:**
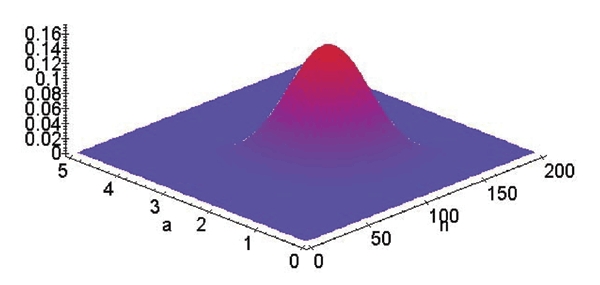
The joint posterior distribution of the attack size and time of attack. Based on a total of 23 cases at the end of 5 days since the initial case was observed. The marginal distributions of Figures 2 and 3 are derived from this joint distribution.

The joint prior distribution of *N* and *A* under the stated assumptions is shown in Figure A1; Figure A2 displays the joint posterior distribution for *N* and *A* after a total of 23 cases have been observed by the end of 5 days after the first case was reported (Figure 1). Results similar to those from Figures 1 to 3 and Figure A2 are obtained if the attack size itself is assumed to be uniformly distributed from 0 to 10,000 for this example, but we believe the log-uniform prior is more sensible in that the primary a priori ignorance regards the order of magnitude of the attack size.
